# Intravenous iron for the treatment of iron deficiency in adults with cystic fibrosis: a prospective observational cohort study

**DOI:** 10.1183/13993003.00838-2025

**Published:** 2025-11-06

**Authors:** Nick P. Talbot, Matilda Downs, Jennifer Cane, Sandi Yen, Goran Mohammad, Alison Gates, Joanna Snowball, Magda Laskawiec-Szkonter, Melissa Dobson, Samira Lakhal-Littleton, Jethro S. Johnson, Najib M. Rahman, Stephen Gerry, Stephen J. Chapman, William G. Flight

**Affiliations:** 1 Department of Physiology, Anatomy and Genetics, University of Oxford, Oxford, UK; 2 Oxford Adult Cystic Fibrosis Centre, Oxford University Hospitals NHS Foundation Trust, Oxford, UK; 3 Nuffield Department of Clinical Medicine, University of Oxford, Oxford, UK; 4 Oxford Centre for Microbiome Studies, Kennedy Institute of Rheumatology, Nuffield Department of Orthopaedics, Rheumatology, and Musculoskeletal Sciences, University of Oxford, Oxford, UK; 5 Centre for Translational Medicine and Therapeutics, The William Harvey Research Institute, Faculty of Medicine and Dentistry, Queen Mary University of London, London, UK; 6 Oxford NIHR Biomedical Research Centre, University of Oxford, Oxford, UK; 7 Centre for Statistics in Medicine, Nuffield Department of Orthopaedics, Rheumatology, and Musculoskeletal Sciences, University of Oxford, Oxford, UK

## Abstract

Iron deficiency is common in chronic cardiorespiratory disease. In patients with heart failure and iron deficiency, intravenous iron improves exercise capacity and quality of life [1]. Similar benefits have been reported in patients with pulmonary hypertension [2] and COPD [3]. The mechanism remains unclear, but in addition to its importance in erythropoiesis, iron availability influences the hypoxia-inducible factor (HIF) transcriptional pathway and modulates physiological responses to hypoxia, particularly within the pulmonary circulation [4].

## Shareable abstract

**In this pilot study in 20 adults with cystic fibrosis and iron deficiency, treatment with intravenous iron improved blood haemoglobin concentration and exercise capacity, without any adverse effect on clinical or laboratory markers of infection** https://bit.ly/47eQKkR

*To the Editor:*

Iron deficiency is common in chronic cardiorespiratory disease. In patients with heart failure and iron deficiency, intravenous iron improves exercise capacity and quality of life [1]. Similar benefits have been reported in patients with pulmonary hypertension [2] and COPD [3]. The mechanism remains unclear, but in addition to its importance in erythropoiesis, iron availability influences the hypoxia-inducible factor (HIF) transcriptional pathway and modulates physiological responses to hypoxia, particularly within the pulmonary circulation [4].

In people with cystic fibrosis (CF), mutation of the cystic fibrosis transmembrane conductance regulator (CFTR) gene leads to defective chloride transport [5]. In the lungs, this causes bronchiectasis and chronic bacterial infection. In the gut, it leads to malabsorption and nutritional deficiency. Iron deficiency is present in up to 60% of people with CF, including those on CFTR modulators [6]. However, it often goes untreated, due to concerns about gastrointestinal upset with oral iron, or the risk of infection with intravenous iron. In particular, the airways of people with CF are often colonised with iron-dependent bacteria such as *Pseudomonas aeruginosa*, and it has been suggested that increasing iron availability could encourage bacterial growth and worsen respiratory infection [7].

We conducted a single-centre open-label pilot prospective observational cohort study in adults with CF and iron deficiency, defined as ferritin ≤15 mg·L^−1^ or transferrin saturation ≤16% within the previous 4 months. Exclusion criteria included pregnancy or breastfeeding, organ transplantation, liver failure, current or recent iron supplementation, and active non-tuberculous mycobacterial pulmonary disease. The study was approved by the South Central Hampshire-A Research Ethics Committee, and was registered at clinicaltrials.gov (NCT03632525). Participants provided written informed consent.

20 patients (mean±sd age 30.1±10.2 years; 50% female) were recruited between March 2019 and March 2020 during routine clinical care in the Oxford Adult Cystic Fibrosis Centre. The recruitment target was predefined, but as a pilot study, no formal power calculation was performed. At baseline, 50% had chronic *Pseudomonas aeruginosa* infection and 95% were pancreatic insufficient. All were iron deficient, but only 40% were anaemic, and the majority would not have received intravenous iron as part of routine care at our centre at the time of the study, due to uncertainty about the risk–benefit balance. In relation to genotype, 55% of participants were homozygous for *F508del* and 35% were heterozygous for *F508del.* No participants were taking CFTR modulator therapy at baseline, but tezacaftor/ivacaftor was commenced in six participants between weeks 8 and 16.

Participants attended four study visits (0, 4, 8 and 16 weeks). At each visit they performed spirometry, provided venous blood and sputum samples, performed a modified shuttle walk test [8], and completed quality of life questionnaires. After these assessments at the week 4 visit, all participants received an intravenous infusion of 20 mg·kg^−1^ ferric carboxymaltose (FCM; Ferinject, Vifor Pharma UK), up to a maximum of 1000 mg if the haemoglobin concentration was <14 g·L^−1^, or 500 mg if ≥14 g·L^−1^. The FCM was given in 250 mL 0.9% saline over 15–30 min.

The primary outcome was a within-patient comparison of the incidence of new infective events in the 4 weeks before and after iron. The definition of a new infective event is provided in table 1. A key secondary outcome was a comparison of the incidence of infective events in the 12 weeks before and after iron (by conducting a retrospective review of the clinical notes). Other clinical and laboratory outcomes are summarised in table 1.

**TABLE 1:** Trial outcomes

Primary outcome	4 weeks before iron	4 weeks after iron	p-value^#^
**Total number experiencing new infective event^¶^**	5 (25%)	5 (25%)	>0.9
New microbiological isolate	3 (15%)	2 (10%)	>0.9
Clinical infection requiring *i.v.* antibiotics	2 (10%)	3 (15%)	>0.9
Admission to hospital for infection-related reason	1 (5%)	2 (10%)	>0.9
Unexplained fall in lung function (>10% FEV_1_)	1 (5%)	0 (0%)	>0.9

Adverse events^+^	4 weeks before iron	4 weeks after iron	
**Total number of adverse events**	35	44	
**Patients experiencing at least one:**			
Adverse event	16 (80%)	17 (85%)	
Serious adverse event	1 (5%)	2 (10%)	

Other infection-related outcomes	12 weeks before iron	12 weeks after iron	p-value^#^
**Total number experiencing new infective event**	11 (55%)	11 (55%)	>0.9
***i.v.* antibiotic days**	7.3±1.9	8.5±2.1	>0.6
**Oral antibiotic days**	8.7±2.5	8.1±1.9	>0.7

Other clinical outcomes	Before iron^§^		After iron^§^		p-value^#^
**Modified shuttle walk distance^ƒ^** **(m)**	892±60		955±59		<0.01
**FEV_1_^##^** **(% pred)**	63±5		63±5		>0.4
**Body mass index (kg·m^−2^)**	23±1		23±1		>0.5
**Systolic pulmonary artery pressure^¶¶^** **(mmHg)**	26±2		25±2		>0.1
**Short Form-36 questionnaire^++^**					
Physical functioning score	75±5		74±6		>0.5
Energy/fatigue score	44±5		50±5		>0.1
**Cystic Fibrosis Questionnaire-Revised^++^**					
Physical functioning score	64±6		64±6		>0.7
Vitality score	49±4		53±5		>0.3

Bloods indices of iron status^§§^	Baseline	Week 4	Week 8	Week 16	p-value^#^
**Haemoglobin (g·L^−1^)**	124±5	125±5	136±3	140±3	<0.001
**Mean cell volume (fL)**	79±2	79±2	84±1	86±1	<0.001
**Ferritin (µg·L^−1^)**	12±2	13±2	194±46	84±15	<0.001
**Transferrin (g·L^−1^)**	3.4±0.1	3.5±0.1	2.5±0.1	2.9±0.1	<0.001
**Serum iron (µg·L^−1^)**	9±1	8±1	14±1	14±2	<0.001
**Soluble transferrin receptor (nmol·L^−1^)**	66±13	73±16	38±6	31±5	<0.001
**Hepcidin (ng·mL^−1^)**	2.6±0.9	2.7±0.6	15.0±3.0	11.4±2.3	<0.001
**Erythropoietin (mIU·mL^−1^)**	54±19	56±21	13±2	16±4	<0.04
**Interleukin-6 (pg·mL^−1^)**	4.6±1.0	5.8±1.0	4.4±0.8	5.8±1.0	>0.7
**C-reactive protein (mg·L^−1^)**	7.2±1.6	10.3±4.2	8.1±2.3	6.6±1.4	>0.4

Sputum microbiome analysis^ƒƒ^	Baseline	Week 4	Week 8	Week 16	p-value^#^
**Shannon's microbiological diversity index**	10.6±2.3	12.7±3.2	9.2±2.7	10.4±3.2	>0.9

Values are given as mean±sem, unless otherwise indicated. 28 patients were identified as eligible based on clinical blood tests, of whom one was ineligible on repeat testing, one could not be contacted, and six declined participation. ^#^: for the primary outcome, and the incidence of infective events in the 12 weeks before and after iron, McNemer's test was used. For the effects of iron on microbiological diversity, the Friedman test was used. For other comparisons, t-tests were used. ^¶^: new infective events were defined as one or more of: 1) new microbiological isolate on sputum culture, not identified within previous 12 months, 2) an infection requiring *i.v.* antibiotics, 3) admission to hospital for an infection-related reason, or 4) significant deterioration in lung function (>10% fall in forced expiratory volume in 1 s (FEV_1_) relative to previous visit), not otherwise explained. The need for *i.v.* antibiotics and the primary reason for admission were judged by the patient's routine clinical team. ^+^: there were no unexpected/reportable serious adverse events during the trial, and no patient withdrawals. Administration of ferric carboxymaltose was generally well-tolerated, with only one participant experiencing a mild–moderate hypersensitivity reaction (rash and chest tightness) at the time of the infusion, which did not result in admission. The serious adverse events in the 4 weeks before and after iron were admissions for treatment of pulmonary exacerbations of cystic fibrosis. A further six serious adverse events occurred between weeks 8 and 16, four of which were admissions for pulmonary exacerbation. In addition, one patient was admitted with distal intestinal obstruction syndrome and another with haemoptysis; neither was felt to be related to iron. ^§^: “before iron” refers to the average of measurements made at baseline and week 4, where available, and “after iron” refers to the average of measurements made at weeks 8 and 16, where available. ^ƒ^: 16 participants (80%) performed at least one shuttle walk test before and one after iron. Four patients declined the shuttle walk test at every visit, and five participants declined at a single visit. ^##^: spirometry was available at all visits for 17 participants (85%). One participant declined spirometry at all time points (due to a history of haemoptysis), one other participant declined at a single time point, and one visit was undertaken remotely. Predicted values were based on reference equations from the Global Lung Function Initiative. ^¶¶^: systolic pulmonary artery pressure was an exploratory outcome, estimated by echocardiographic measurement of the tricuspid value regurgitant jet velocity, assuming a right atrial of pressure of 5 mmHg. Measurement of systolic pulmonary artery pressure was possible during at least one visit before and after iron in 11 participants. ^++^: all participants completed the Short Form-36 and Cystic Fibrosis Questionnaire-Revised questionnaires at all visits. The results from selected domains are provided in the table, but there was no significant effect of iron on any domain of either questionnaire. ^§§^: blood was available for all participants at all time-points, other than the final visit for the final participant, which was undertaken remotely due to the onset of the COVID-19 pandemic. Plasma was stored at −80°C for ELISA analysis (R&D systems Quantikine assay for erythropoietin and interleukin-6, DRG assay for soluble transferrin receptor and hepcidin). Other indices were measured on the day of the visit *via* the clinical laboratory at the John Radcliffe Hospital, Oxford, UK. ^ƒƒ^: sputum was obtained from 16 patients (80%) at all visits, and from 19 patients (95%) in total. Samples were submitted for microscopy and standard bacterial, fungal and mycobacterial culture *via* the clinical laboratory. In addition, whole sputum plugs were stored at −80°C for subsequent DNA extraction using FASTDNA SPIN kit for Soil (MP Biomedicals). For bacterial 16S rRNA gene amplicon sequencing, the variable V3 and V4 regions of the 16S rRNA gene were amplified from genomic DNA and amplicons were attached with indices and Illumina sequencing adapters using the Nextera XT index kit. The 16S amplicon libraries were pooled and sequenced in an Illumina MiSeq v3 flowcell as 300 base pair paired-end reads. Library preparation and sequencing was performed at the Oxford Genomics Centre. A community DNA standard (Zymobiomics, D6305) and the extraction negative control were included as sequencing positive and negative controls, respectively.

There was no difference in the incidence of new infective events in the 4 weeks before and after iron, and no difference for the 12 weeks before and after iron. Antibiotic use was similar across these periods and there was no apparent impact of iron on sputum microbiological diversity. These findings are in keeping with the safety of intravenous iron in other populations vulnerable to infection, including patients on immunosuppressive therapy [9] or in the critical care setting [10]. Our results also accord with those of a previous study of oral iron supplementation in adults with CF and iron deficiency, in which 6 weeks of oral ferrous sulphate did not alter sputum microbiological diversity [11].

Iron led to a substantial and sustained increase in iron availability, with a corresponding increase in haemoglobin. The cause of iron deficiency in people with CF is unclear, but candidate mechanisms include blood loss into airways, menstrual loss, malabsorption, and so-called “functional” iron deficiency, in which infection and/or inflammation inhibits iron uptake in the gut and causes sequestration within reticuloendothelial macrophages. In our study, hepcidin levels were suppressed at baseline, with elevated serum soluble transferrin receptor levels. This pattern is characteristic of absolute, rather than functional, iron deficiency, and the elevated erythropoietin and low haemoglobin concentration at baseline suggest iron-restricted erythropoiesis. In other settings, hepcidin and erythropoietin are used to identify patients likely to respond to iron supplementation, which might also prove helpful in those with CF [12].

An important finding in the current study is the apparent increase in exercise capacity. Exercise has numerous benefits in people with CF, including improved airway clearance and bone protection. If sustained, improved exercise capacity seems likely to improve long term clinical outcomes. In the current trial, it may result from the increased haemoglobin concentration after iron, but similar increases are independent of haemoglobin in patients with other cardiorespiratory conditions [1–3]. Possible mechanisms include a direct impact on cardiac function or a prolonged reduction in the pulmonary vascular sensitivity to hypoxia, which is known to occur following intravenous iron [13]. There was no clear fall in the systolic pulmonary artery pressure after iron in the current study, but measurements were possible in only around half the participants, and were made only at rest.

There was no effect of iron on any domain of the CFQ-R (Cystic Fibrosis Questionnaire-Revised) or SF-36 (Short Form-36) quality of life measures, but the study lacked statistical power for these outcomes. Of note, only one participant (5%) reported fatigue during the 4 weeks after iron, compared with four participants (20%) in the 4 weeks before iron. In contrast, five participants (25%) experienced transient flu-like symptoms, including myalgia, in the days following iron. One possible cause is hypophosphataemia, which is common after FCM, and can cause malaise and/or myalgia [14].

This study has a number of limitations. First, as a pilot study in 20 patients with a non-randomised design, it is not possible to attribute changes in outcomes definitively to iron administration. However, in the absence of any other prospective data on the safety of intravenous iron in people with CF, our results do assuage concerns about the risk of worsening infection-related outcomes raised by previous case series [7]. Second, although our primary outcome is a broad, multifaceted measure of infective status, it has not been previously validated. Third, our study was relatively short, and we cannot exclude adverse or beneficial effects beyond the 12-week follow up period. In this context, iron deficiency has recently been shown to be associated with impaired adaptive immunity and vaccine efficiency [15]. If this were to be the case for people with CF, there may be long-lasting positive effects of treating iron deficiency on the incidence of respiratory infection.

Finally, this study was conducted before the widespread use of CFTR modulator therapy in people with CF. The introduction of such therapy in six patients between weeks 8 and 16 could not have influenced the primary outcome, but could have influenced some secondary outcomes. In relation to the shuttle walk distance, the increase after iron remained significant when these six participants were excluded from the analysis.

In summary, in this pilot study, administration of intravenous iron in adults with CF and iron deficiency had no adverse impact on infective status, but was associated with a sustained improvement in iron status, correction of anaemia and improved exercise capacity. This preliminary result paves the way for a larger randomised controlled trial of intravenous iron supplementation in people with CF, focused on the potential for longer-term benefits. If confirmed in larger studies, our results would also have implications for the use of intravenous iron in other groups at high risk of infection, including those with non-CF bronchiectasis or patients in the critical care setting.

## Shareable PDF

10.1183/13993003.00838-2025.Shareable1 This PDF extract can be shared freely online.
Shareable PDF

ERJ-00838-2025.Shareable

## Data Availability

Individual de-identified participant data can be made available for scientific purposes upon submission of a reasonable request to the corresponding author.

## References

[1] Anker SD, Comin-Colet J, Filippatos G, et al. Ferric carboxymaltose in patients with heart failure and iron deficiency. N Engl J Med 2009; 361: 2436–2448. doi:10.1056/NEJMoa0908355 19920054

[2] Ruiter G, Manders E, Happe CM, et al. Intravenous iron therapy in patients with idiopathic pulmonary arterial hypertension and iron deficiency. Pulm Circ 2015; 5: 466–472. doi:10.1086/682217 26401247 PMC4556497

[3] Santer P, McGahey A, Frise MC, et al. Intravenous iron and chronic obstructive pulmonary disease: a randomised controlled trial. BMJ Open Respir Res 2020; 7: e000577. doi:10.1136/bmjresp-2020-000577 PMC7311010 32565444

[4] Smith TG, Talbot NP, Privat C, et al. Effects of iron supplementation and depletion on hypoxic pulmonary hypertension: two randomized controlled trials. JAMA 2009; 302: 1444–1450. doi:10.1001/jama.2009.1404 19809026

[5] Shteinberg M, Haq IJ, Polineni D, et al. Cystic fibrosis. Lancet 2021; 397: 2195–2211. doi:10.1016/S0140-6736(20)32542-3 34090606

[6] Jia S, Wang Y, Ross MH, et al. Association between CFTR modulators and changes in iron deficiency markers in cystic fibrosis. J Cyst Fibros 2024; 23: 878–884. doi:10.1016/j.jcf.2024.03.002 38490920 PMC11399321

[7] Hoo ZH, Wildman MJ. Intravenous iron among cystic fibrosis patients. J Cyst Fibros 2012; 11: 560–562. doi:10.1016/j.jcf.2012.05.002 22717534

[8] Bradley J, Howard J, Wallace E, et al. Validity of a modified shuttle test in adult cystic fibrosis. Thorax 1999; 54: 437–439. doi:10.1136/thx.54.5.437 10212110 PMC1763768

[9] Keating GM. Ferric carboxymaltose: a review of its use in iron deficiency. Drugs 2015; 75: 101–127. doi:10.1007/s40265-014-0332-3 25428711

[10] Litton E, Baker S, Erber WN, et al. Intravenous iron or placebo for anaemia in intensive care: the IRONMAN multicentre randomized blinded trial: a randomized trial of IV iron in critical illness. Intensive Care Med 2016; 42: 1715–1722. doi:10.1007/s00134-016-4465-6 27686346

[11] Gifford AH, Alexandru DM, Li Z, et al. Iron supplementation does not worsen respiratory health or alter the sputum microbiome in cystic fibrosis. J Cyst Fibros 2014; 13: 311–318. doi:10.1016/j.jcf.2013.11.004 24332997 PMC3972336

[12] Shah A, Wray K, James T, et al. Serum hepcidin potentially identifies iron deficiency in survivors of critical illness at the time of hospital discharge. Br J Haematol 2019; 184: 279–281. doi:10.1111/bjh.15067 29363744

[13] Bart NK, Curtis MK, Cheng HY, et al. Elevation of iron storage in humans attenuates the pulmonary vascular response to hypoxia. J Appl Physiol (1985) 2016; 121: 537–544. doi:10.1152/japplphysiol.00032.2016 27418684 PMC5007321

[14] Wolf M, Rubin J, Achebe M, et al. Effects of iron isomaltoside *vs* ferric carboxymaltose on hypophosphatemia in iron-deficiency anemia: two randomized clinical trials. JAMA 2020; 323: 432–443. doi:10.1001/jama.2019.22450 32016310 PMC7042864

[15] Stoffel NU, Drakesmith H. Effects of iron status on adaptive immunity and vaccine efficacy: a review. Adv Nutr 2024; 15: 100238. doi:10.1016/j.advnut.2024.100238 38729263 PMC11251406

