# Supplementary material for: Intravenous iron for the treatment of iron deficiency in adults with cystic fibrosis: a prospective observational cohort study
Source: Eur Respir J. 2025 Nov 6;66(5):2500838. doi: 10.1183/13993003.00838-2025 (PMC12591133; doi:10.1183/13993003.00838-2025)
Supplement: Supplementary file 1 [file ERJ-00838-2025.Shareable.pdf]

SHAREABLE PDF

Intravenous iron for the treatment of iron deficiency in adults with cystic fibrosis: a prospective observational cohort study

Nick P. Talbot [1,2,3], Matilda Downs [1,3], Jennifer Cane [3], Sandi Yen [4], Goran Mohammad [1,5], Alison Gates [2], Joanna Snowball [2], Magda Laskawiec-Szkonter [3], Melissa Dobson [3], Samira Lakhal-Littleton [1], Jethro S. Johnson [4], Najib M. Rahman [3,6], Stephen Gerry [7], Stephen J. Chapman [2] and William G. Flight [2]

[1]Department of Physiology, Anatomy and Genetics, University of Oxford, Oxford, UK. [2]Oxford Adult Cystic Fibrosis Centre, Oxford University Hospitals NHS Foundation Trust, Oxford, UK. [3]Nuffield Department of Clinical Medicine, University of Oxford, Oxford, UK. [4]Oxford Centre for Microbiome Studies, Kennedy Institute of Rheumatology, Nuffield Department of Orthopaedics, Rheumatology, and Musculoskeletal Sciences, University of Oxford, Oxford, UK. [5]Centre for Translational Medicine and Therapeutics, The William Harvey Research Institute, Faculty of Medicine and Dentistry, Queen Mary University of London, London, UK. [6]Oxford NIHR Biomedical Research Centre, University of Oxford, Oxford, UK. [7]Centre for Statistics in Medicine, Nuffield Department of Orthopaedics, Rheumatology, and Musculoskeletal Sciences, University of Oxford, Oxford, UK.

Corresponding author: Nick P. Talbot (nick.talbot@dpag.ox.ac.uk)

Shareable abstract (@ERSpublications)
In this pilot study in 20 adults with cystic fibrosis and iron deficiency, treatment with intravenous iron improved blood haemoglobin concentration and exercise capacity, without any adverse effect on clinical or laboratory markers of infection https://bit.ly/47eQKkR

Cite this article as: Talbot NP, Downs M, Cane J, *et al*. Intravenous iron for the treatment of iron deficiency in adults with cystic fibrosis: a prospective observational cohort study. *Eur Respir J* 2025; 66: 2500838 [DOI: 10.1183/13993003.00838-2025].

This PDF extract can be shared freely online.

Copyright ©The authors 2025

This version is distributed under the terms of the Creative Commons Attribution Licence 4.0.

Received: 25 June 2024
Accepted: 18 Aug 2025

To the Editor:

Iron deficiency is common in chronic cardiorespiratory disease. In patients with heart failure and iron deficiency, intravenous iron improves exercise capacity and quality of life [1]. Similar benefits have been reported in patients with pulmonary hypertension [2] and COPD [3]. The mechanism remains unclear, but in addition to its importance in erythropoiesis, iron availability influences the hypoxia-inducible factor (HIF) transcriptional pathway and modulates physiological responses to hypoxia, particularly within the pulmonary circulation [4].
